# Non-Invasive Monitoring of Functional State of Articular Cartilage Tissue with Label-Free Unsupervised Hyperspectral Imaging

**DOI:** 10.1038/s41598-019-40942-7

**Published:** 2019-03-13

**Authors:** Saabah B. Mahbub, Anna Guller, Jared M. Campbell, Ayad G. Anwer, Martin E. Gosnell, Graham Vesey, Ewa M. Goldys

**Affiliations:** 1 ARC Centre of Excellence for Nanoscale Biophotonics, Macquarie University, Sydney, NSW Australia; 2 Graduate School of Biomedical Engineering, UNSW Australia, Sydney, NSW 2052 Australia; 3 Quantitative Pty Ltd, 116-118 Great Western Highway, Mt. Victoria, NSW 2786 Australia; 4 Regeneus Pty Ltd, 25 Bridge Street, Pymble, NSW 2073 Australia

## Abstract

Damage and degradation of articular cartilage leads to severe pain and loss of mobility. The development of new therapies for cartilage regeneration for monitoring their effect requires further study of cartilage, ideally at a molecular level and in a minimally invasive way. Hyperspectral microscopy is a novel technology which utilises endogenous fluorophores to non-invasively assess the molecular composition of cells and tissue. In this study, we applied hyperspectral microscopy to healthy bovine articular cartilage and osteoarthritic human articular cartilage to investigate its capacity to generate informative molecular data and characterise disease state and treatment effects. We successfully demonstrated label-free fluorescence identification of collagen type I and II – isolated in cartilage here for the first time and the co-enzymes free NADH and FAD which together give the optical redox ratio that is an important measure of metabolic activity. The intracellular composition of chondrocytes was also examined. Differences were observed in the molecular ratios within the superficial and transitional zones of the articular cartilage which appeared to be influenced by disease state and treatment. These findings show that hyperspectral microscopy could be useful for investigating the molecular underpinnings of articular cartilage degradation and repair. As it is non-invasive and non-destructive, samples can be repeatedly assessed over time, enabling true time-course experiments with in-depth molecular data. Additionally, there is potential for the hyperspectral approach to be adapted for patient examination to allow the investigation of cartilage state. This could be of advantage for assessment and diagnosis as well as treatment monitoring.

## Introduction

Damage and degradation of articular cartilage, occurring in osteoarthritis, trauma and other joint conditions, leads to severe pain and reduced mobility^1,2^. Current treatment strategies are mainly focused on controlling inflammation in soft tissue, while options for restoration of the cartilage are limited. Due to the absence of blood vessels cartilage grows and repairs more slowly than other tissues making cartilage regeneration difficult^3^. Current treatments beyond anti-inflammatories^4^ include intra-articular injections of hyaluronan^5^ or preparations based on adult mesenchymal stem cells (MSC)^6,7^. MSC preparations have been shown to aid cartilage regeneration^7–9^, whereas the effects of other therapies are restricted to symptom management. Some MSC preparation therapies may also contain hyaluronan^10,11^. Cartilage regeneration success is manifested through a reduction in the size of cartilage defects and the formation of new hyaline-like cartilage^12^. The latter can only be confirmed by histopathological examination, necessitating the collection of tissue samples. The development of new therapies for cartilage regeneration, as well as techniques for monitoring their effect will require further study of cartilage, ideally on a molecular level and in a minimally invasive way.

The condition of cartilage and/or its damage can be characterised by common medical imaging modalities such as computed tomography^13^, high resolution microcomputed tomography^14^, or magnetic resonance imaging^15^. None of these standard techniques have molecular sensitivity. A standard histological assessment of cartilage requires tissue sampling, time-consuming preparation and does not provide highly specific molecular information. Immunohistochemistry can identify collagen type I and II in cartilage, and this approach has been used in the literature to explore cartilage regeneration study^16^. However, these conventional methods are laborious, costly, invasive and can only provide a snapshot of a tissue structure and its functional state on a sample-by-sample basis.

Continuous monitoring and *in situ* assessment of cartilage structure and functional state requires a minimally-invasive method, preferably without a biopsy. As a first step towards addressing this problem we have explored the potential of label-free multispectral imaging of endogenous tissue fluorescence^17^ to characterise the molecular composition, structure and functional status of articular cartilage. We have applied this methodology to examine the native distribution of endogenous tissue fluorophores in intact articular cartilage, and subsequently demonstrated the potential of our methodology to characterise the effects of an experimental treatment of osteoarthritic (OA) cartilage (based on secretions from adipose-derived human MSCs) performed *ex vivo*.

Our approach is based on multispectral imaging of tissue autofluorescence followed by unmixing of the fluorescence signals of individual compounds native to cartilage, identified using a previously reported unmixing approach^17^. These compounds, most notably nicotinamide adenine dinucleotide (NADH) and flavin adenine dinucleotide (FAD), can provide informative signatures of cellular metabolic activity^18–20^. A related parameter, called the optical redox ratio (RR, the ratio of the intensities of FAD and NADH fluorescent signals), is directly associated with cell metabolism^20–22^.

Cartilage consists of cells called chondrocytes^23^ surrounded by specialized extracellular matrix (ECM). The chondrocytes produce the ECM components, including collagens, aggrecans, small proteoglycans, and elastic fibers^24^. Collagens, especially collagen type II significantly contribute to the strong intrinsic fluorescence of cartilage^25,26^.

Four different zones (superficial, transitional, deep and calcified) can be identified in a cross-section of articular cartilage tissue between its surface and the subchondral bone (shown in Supplementary Fig. S1 and Supplementary Section 1). The superficial zone, which has a smooth outer surface, facilitates bone gliding. It forms 10% to 20% of the thickness of articular cartilage (~ 0.25 mm, in bovine knee joints) and has the highest collagen contents of the tissue zones^27^. The underlying transitional (middle) zone comprises a further 40% to 60% of the thickness of the articular cartilage (0.3–1 mm in bovine joints). The main components of the ECM in the transitional zone are collagen (about 60% of the dry weight) and aggrecans^27^. Chondrocytes in this layer are more rounded than the superficial zone^27^. The composition of the ECM differs between the superficial and transitional zones of cartilage. In healthy articular cartilage collagen I is found mostly in the superficial layer and, in moderate amounts, the transitional layer^28^, while collagen type II forms up to 90–95% of the total collagen content of the transitional zone^25–27^. Other collagen types (IV, V, VI, IX, and XI) contribute only a minor fraction, but they play a major role in forming and stabilizing collagen II fibrils^27^. In patients with OA, the zonal structure of cartilage is compromised. The surface of the OA cartilage becomes uneven, with variable depth of defects, from a few micron defects in the superficial layer up to full-thickness lesions, reaching the subchondral bone^29^.

Healthy hyaline articular cartilage is not vascularized and this has an effect of chondrocyte metabolism. The superficial layer receives the oxygen and nutrients from the synovial liquid of the articular cavity, and the deeper parts of the cartilage are supplied from the highly vascularized subchondral bone. These features limit the regenerative potential of cartilage in the superficial and transitional zones. Accelerating cartilage regeneration by stimulating articular chondrocytes is a significant clinical objective. In this work we have demonstrated label-free fluorescence identification of collagen type I and II in samples of excised articular cartilage for the first time. This is also the first report of label-free characterization of the functional state of chondrocyte, without any staining, using standard single photon fluorescence. This simple approach is more suitable for clinical adoption compared to the alternative approach of second harmonic generation (SHG) imaging^30,31^. We show that hyperspectral imaging can serve as a sensitive tool to monitor the functional state of articular cartilage and to non-invasively examine the tissue response to treatments applied to the cartilage surface.

## Methods

### Overview of imaging methods

We used a fluorescence microscope (Olympus iX71™) with a 40× water U12™ series objective, with a range of selected bands of excitation wavelengths to generate a total of 18 specific channels to measure single photon-excited emission of biological samples (detailed image acquisition methods are given in Supplementary Material, Section 2). All images were captured by an Andor iXON™ camera (EMCCD, iXON 885 DU, Andor Technology Ltd., UK) operated below −65 °C to reduce sensor-induced noise. We used image acquisition times of up to 5 seconds per channel, with multiple averaging (typically 3–5 times) to optimise image quality.

Due to computational complexity the unmixing analysis was carried out using sparsified images wherein representative small areas (total ~4000 pixels per area) were selected and analysed (details of image processing are given in Supplementary Materials Section 3 and Supplementary Table S1). In thin samples we selected two such areas within the chondrocytes and two areas containing ECM only. The unmixing results were then weighted to reflect the overall ratio of chondrocyte to ECM areas in the hyaline cartilage (~8.6% for the superficial layer and ~6.7% for the transitional layer). In thick samples where chondrocytes were not visible, the areas were randomly selected. After that, the Robust Dependent Component Analysis^17,32^ (RoDECA) algorithm was used for identifying the dominant native fluorophores and their corresponding abundance (Details are in Supplementary Material, Sections 4 and 5).

**Figure 1:**
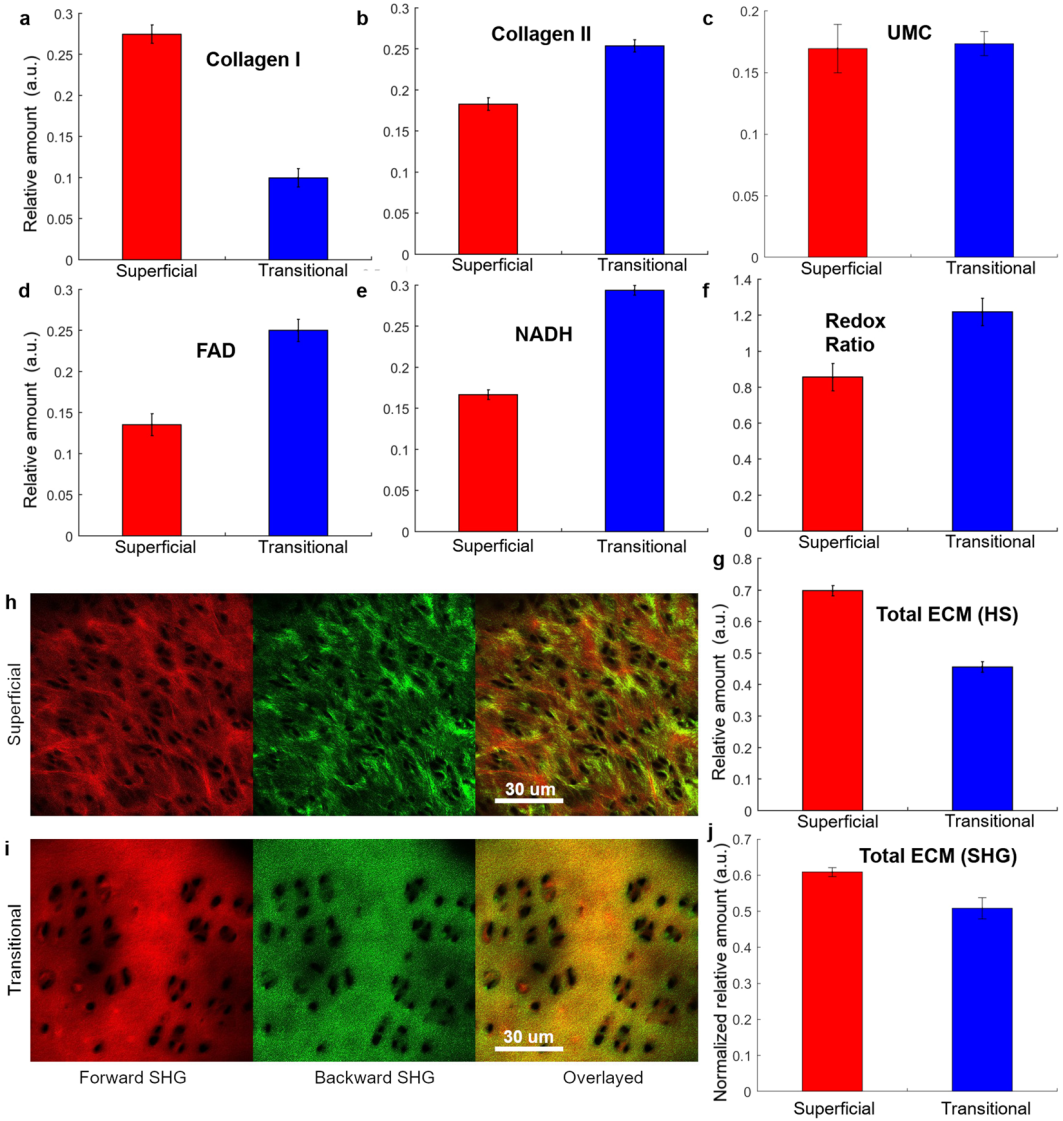
Mean abundances of main autofluorescent components in superficial (red) and transitional (blue) layers of intact bovine articular cartilage (sample set 1–8). The bars represent the standard error from multiple samples (Supplementary Table S1). The extracted mean abundances of (**a**) collagen I, (**b**) collagen II, (**c**) unknown ECM component, UMC, (**d**) FAD, (**e**) NADH, (**f**) mean value of RR (FAD/NADH) and (**g**) total abundance of ECM obtained from hyperspectral imaging, calculated here as the sum of abundances of collagen I, collagen II and the fifth undefined fluorophore, likely to be also collagen or aggrecans. All values are calculated from mean abundances of extracted fluorophores. (**h**,**i**) Forward (first column) and backward scattered (middle column) SHG images for (**h**) superficial (upper row) and (**i**) transitional layers (bottom row) and the overlayed SHG images (third column). (**j**) Normalized mean abundance of total ECM in the superficial (red) and transitional layers (blue) derived from the SHG signal.

For benchmarking, the bovine samples (sample set 7–8, Supplementary Table S1) were imaged using second harmonic generation (SHG) (Leica: TCS-MP-SP5 with 880 nm laser) and the SHG signal (forward and backward) was detected at 440 nm. Z-stacks of each frozen sections (10 µM) describe the total collagen structure and are presented in Fig. 1(h,i). After image filtering (as described in Supplementary Material, Section 2.3), the analysis of the total ECM signal (sum of the mean intensities of the forward and backward channels) was carried out using Matlab™.

### Bovine cartilage – sample preparation

Fragments of bovine cartilage fragments were harvested from the knee joints of commercially available cattle carcasses. The carcases were chilled to 0 to 5 °C^33^ and the complete knee joints were collected within 24 hours post mortem^34^. The knee joints were cut out by sawing 15 cm below and above the joint. The femoral joint was then shaved, scrubbed, and soaked in an iodine bath for a minimum time of 5 minutes and then frozen for later use^35^. The frozen bovine knee joints were thawed at 37 °C for 1–2 hours until they became flexible. Once thawed, the joints were disarticulated in a class II sterile biosafety cabinet and the articular cartilage was tangentially excised from the femoral medial condyle into approximately 1 mm thick slabs. The slabs were then inspected and any slab that contained macroscopically visible signs of defects was discarded. The experiments were done under ethics of Biosafety (AAN300412BHA, Macquarie University) approval.

Next, 10 μm thick cryosections of bovine cartilage were prepared from superficial and transitional layers slicing cartilage blocks (sample set 1–8, Supplementary Table S1). These thinner slices were required to achieve sharp focus and obtain high quality second harmonic generation (SHG) images. In order to provide a clear distinction, the superficial cartilage samples were taken from the first five thin sections (50 µm from the tissue surface), while the samples from the transitional layer were taken from the depth between 300 to 400 μm from the cartilage surface. The cryosections were immediatly mounted onto the glass coverslip and kept on dry ice (−70 °C) before imaging. During hyperspectral imaging, 0.2 ml of Hanks Balanced Salt Solution (HBSS, Life Tech, Australia, 14025076) was added to each coverslip and the temperature was maintained at 37 °C.

### Human cartilage - sample preparation and treatments

Human knee joint samples from two patients (over 45 years, male) with osteoarthritis (OA) who were undergoing total knee replacement surgeries were collected fresh, within 1 hour after the operation. The experiments were carried out in accordance with the approved Human ethics (ref: 5201300753, Macquarie University with informed consent was obtained from all subjects) and Biosafety (AAN300412BHA, Macquarie University). Thick cartilage fragments (diameter 6.45 mm and thickness 0.5 mm) were produced from the medial condyle and soaked in PBS solution. This sample size matches the well size of the 96 well plates where the experimental treatment was carried out. For these thick tissue samples, the focusing problem was addressed as described in Supplementary Materials, Section 7. Thin (10–20 um) tissue slices (Section 2.2) did not present focusing problems.

The cartilage fragments from patient 1 were divided into two groups. The first group was used as a control representing untreated OA articular cartilage (sample set 9–12, Supplementary Table S1). These samples were soaked in a standard cell culture medium (consisting of Dulbecco’s Modified Eagle Medium (DMEM; Invitrogen, USA) supplemented with 10% foetal bovine serum (FBS; Bovogen, Australia) and 1% penicillin-Streptomycin solution from Invitrogen, USA) for 72 hours. The second group (sample set 13–16, Supplementary Table S1) was treated with the same culture medium with a proprietary composition containing secretions from adipose-derived human MSCs (supplied by Regeneus Pty Ltd, Australia) for the same period, and assigned thereafter as “Treatment A”.

The cartilage chips from patient 2 were divided into three groups. The first group was used as a control for OA cartilage (sample set 17–20, Supplementary Table S1) and prepared in the same way as the control samples from patient 1. The second group (sample set 21–24, Supplementary Table S1) was treated as described above for Treatment A. The third group of samples (sample set 25–28, Supplementary Table S1) were treated with the same culture medium containing secretions from adipose-derived human MSCs supplied by Regeneus Pty Ltd., Australia, but with the addition of 6% v/v hyaluronan) for 72 hours; assigned thereafter “Treatment B”. Samples from both patients were imaged and analysed using the same approaches and conditions.

Treatment A and B are based on soluble active factors from MSCs and mirror MSC-based regenerative treatments^8,9^ but do not use cells, which makes them easier to produce, distribute and apply. Treatment B is an altered form of treatment A which includes hyaluronan^10,11^.

## Results

### Hyperspectral analysis and comparison with second harmonic generation imaging

Hyperspectral unmixing was used to assess the relative normalized amount (abundance) of individual native fluorophores in intact articular cartilage^17^. Five fluorophores were unmixed, four of which (collagen I, collagen II, FAD, and free NADH) closely matched the spectral characteristics of reference fluorophores (Section 8 and 9, Supplementary Material). The fifth component, an unknown ECM component, was termed unknown matrix component (UMC). The relative amount of these fluorophores in the superficial and transitional layers of intact bovine cartilage are shown in Fig. 1 (more details in Supplementary Fig. S7a).

As can be seen from Fig. 1(a–g), the superficial and transitional layers of bovine articular cartilage have different molecular compositions. Our hyperspectral imaging results show that the superficial layer contains more collagen I (Fig. 1a) and less collagen II (Fig. 1b), than the transitional layer. The relative amount of collagen I in the superficial and transitional layers was, respectively, 39.6 ± 7.6% and 22.3 ± 9.5% of the total ECM, while in the case of collagen II it was, respectively 36.5 ± 7.9% and 39.6 ± 6.8% of the total ECM. This ratio reflects the fibrocartilage nature of the superficial layer and hyaline cartilage characteristics of the transitional layer^36^. The unknown ECM component UMC (see Fig. 1(c)) has almost the same relative amount in the transitional and superficial layers (0.17 ± 0.02 a.u. (arbitrary units) vs. 0.16 ± 0.02 a.u). In the superficial layer this component was less abundant than collagen types I and II, while in the transitional layer the relative amount of UMC was comparable to that of collagen II. The spectrum of UMC was similar to the spectra of collagen III and V (Supplementary Fig. S9d). We found that the total ECM relative amount (Fig. 1g) was higher in the superficial than in the transitional layer (0.69 ± 0.01 vs. 0.45 ± 0.01). This is consistent with a higher density of the superficial layer^30^, also apparent from the SHG signal, presented in Fig. 1j, and to a comparatively lower cell density in the superficial and transitional layers (27 ± 4 vs 35 ± 3 per 0.03 mm^2^ area, respectively).

The ECM composition of the intact bovine articular cartilage was statistically significantly associated with the optical RR (FAD/NADH) (Table 1). This correlation is indicative of the different metabolic state (−0.354 and 0.156, p < 0.001) of the chondrocytes at superficial and transitional layers. The accumulation (increased production and/or down-regulation of degradation) of collagen type I by the chondrocytes was associated with decreased accumulation of collagen type II (−0.326 vs. −0.843, p < 0.001) in the superficial and transitional layers respectively. The accumulation of collagen I in the superficial layer was associated with lower levels of NADH and increased of FAD (See Supplementary Table S3 for details of correlations) – resulting in in a higher correlative RR (0.618 vs −0.038). In the transitional layer the accumulation of collagen I was mostly independent of the cellular metabolic state.

**Table 1:** Correlation of bovine cartilages with ECM components and RR.

Intact Bovine	Superficial layer				Transitional layer			
	Col I	Col II	ECM	RR	Col I	Col II	ECM	RR
Collagen I	1.000	−0.326	−0.027	0.618	1.000	−0.843	−0.823	−0.034
Collagen II	−0.326	1.000	0.385	−0.225	−0.843	1.000	0.799	−0.038
ECM	−0.027	0.385	1.000	−0.354	−0.823	0.799	1.000	0.156
RR	0.618	−0.225	−0.354	1.000	−0.034	−0.038	0.156	1.000

The identity of collagen autofluorescence signal was confirmed by SHG microscopy imaging. The SHG signal is not sensitive to collagen type (I, II, III, V, XI)^37–39^, but the ratio between forward and backward SHG signals reflects the organization of total collagen fibrils^30^; hence the higher ECM signal caused by an increased fibril diameter or the thickness of bundles composed of densely packed thinner fibrils^30^. The normalized SHG signal of the ECM for both superficial (in Fig. 1h) and transitional (in Fig. 1i) layers is presented in Fig. 1j. The results confirm that the superficial layer produces a higher SHG signal (0.61 ± 0.01 vs. 0.5 ± 0.01), in analogy to the unmixed total ECM autofluorescence signal in Fig. 1g.

### The ratio of cellular fluorophore components

**Figure 2:**
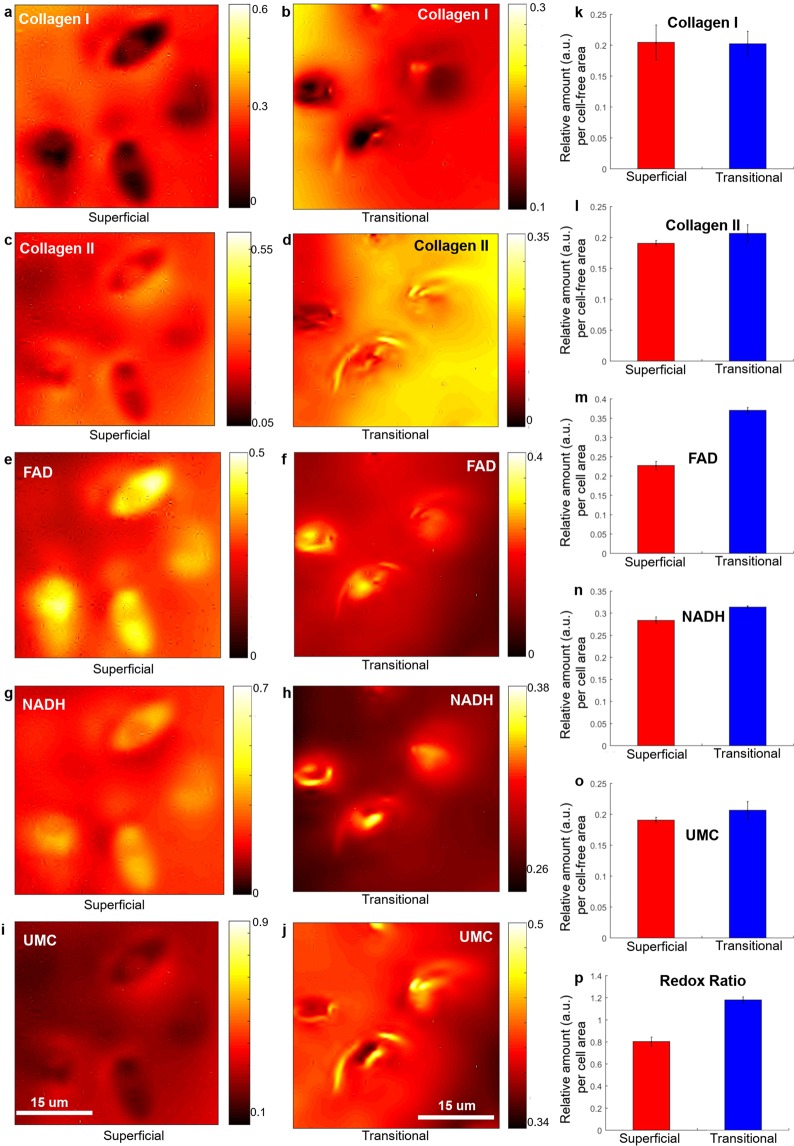
Abundance maps of the extracted fluorescent components in intact bovine articular cartilage; collagen I (**a**,**b**), collagen II (**c**,**d**), FAD (**e**,**f**), NADH (**g**,**h**) and UMC (**i**,**j**) in ECM of superficial (first column, (**a**, **c**, **e**, **g** and **i**)) and transitional (middle column, (**b**, **d**, **f**, **h** and **j**)) layers (sample set 1 for superficial and sample set 2 for transitional layer, as listed in Supplementary Table S1). The abundances per cell area (using manually segmented chondrocytes from the abundance images, positive masking) and per cell-free (negative masking) area are also presented for superficial (red) and transitional (blue) layers. (**k**) Collagen I, (**l**) Collagen II, (**m**) FAD, (**n**) NADH, (**o**) the unknown ECM component, UMC; (**p**) RR (FAD/NADH) per cell area. The error bars are calculated from the analysed cell number (4 cells in superficial layers and 3 cells in transitional layers) for FAD, NADH and RR. Note that scales and colour-bars are different between images to enable legibility.

Figure 1(d–f) shows that the abundance of FAD and NADH, as well as the optical RR, was lower in the superficial layer of the intact bovine articular cartilage compared to the transitional layer. To further interpret these findings, we utilised the ability of hyperspectral analysis to visualise chondrocytes in the thin cartilage samples (Fig. 2a and b for superficial and transitional layers) in order to obtain average cell densities and calculate the average cellular abundance of FAD (Fig. 2m), NADH (Fig. 2n) and the average RR (Fig. 2p) per cell. Although relative amount of NADH was not strongly influenced FAD was significantly lower in chondrocytes from the superficial layer compared to those from the transitional layer (0.22 ± 0.01 vs 0.37 ± 0.01) which resulted in a significant decrease in average cellular RR (0.8 ± 0.02 vs 1.2 ± 0.01) confirming the transition of chondrocytes in the transitional layer from glycolysis to oxidative phosphorylation.

### Detection of the effects of experimental treatment of human osteoarthritic articular cartilage *in vitro*

Having validated the hyperspectral imaging method for non-invasively visualising collagen I and II, NADH and FAD, we applied it to OA human cartilage exposed to experimental treatments to induce cartilage regeneration. The aim of this experiment was to determine the sensitivity of the HS imaging method to detect the changes induced in the articular cartilage under various stimuli (details are in Section 2.4). The same five fluorophores were successfully identified in these samples (sample set 9–28, Supplementary Table S1). Four of those, (collagen I and II, FAD, and free NADH) matched the fluorophores from our reference bank (see Supplementary Fig. S7 for more details).

**Figure 3:**
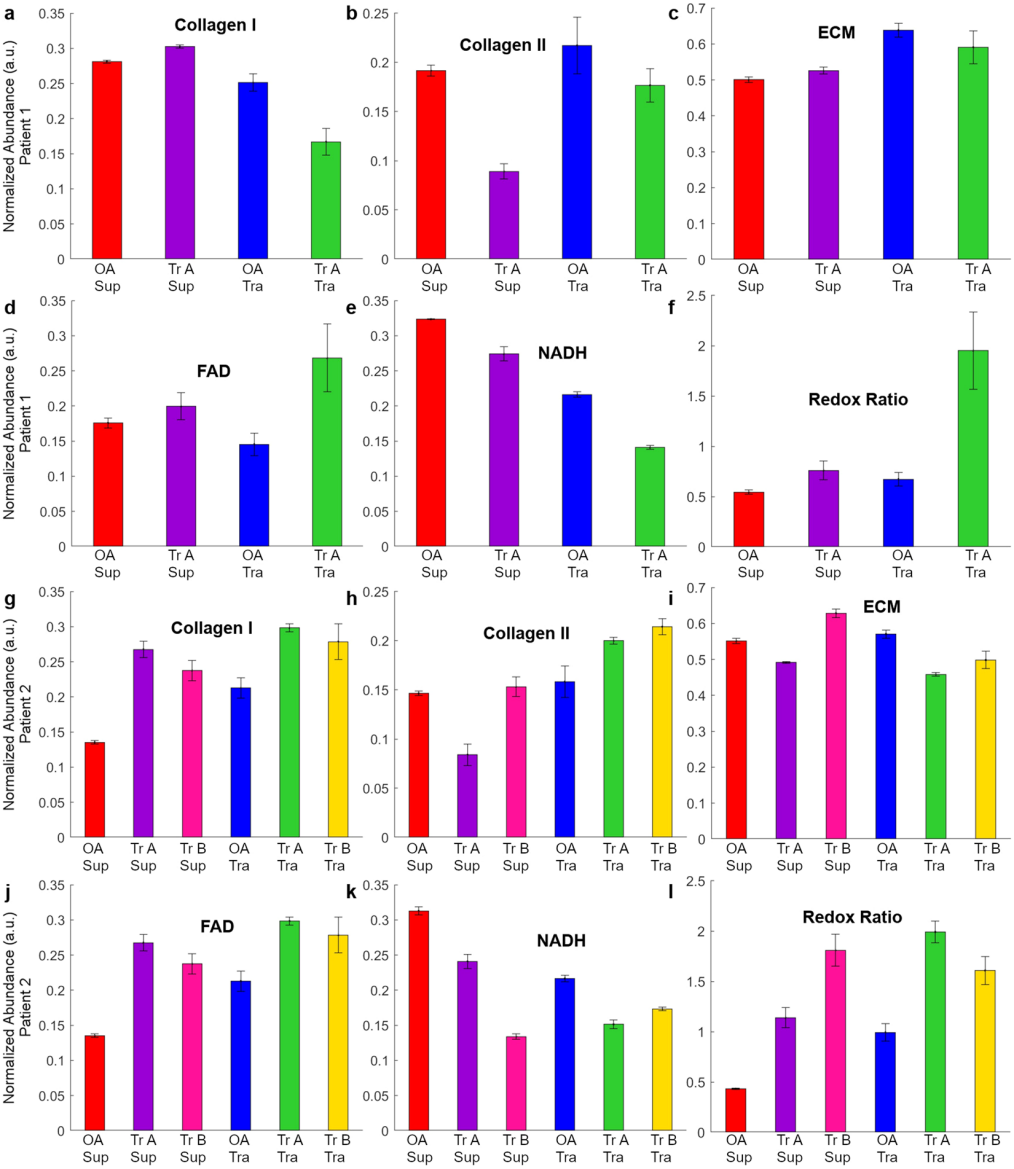
(**a**–**f**) Ratio of the main fluorescent components in ECM of superficial (Sup) and transitional (Tra) layers of human articular cartilage for patient 1. The extracted normalized abundances (**a**) collagen I, (**b**) collagen II, (**c**) ECM (**d**) FAD, (**e**) NADH, and (**f**) RR is presented for patient 1. (**g**–**l**) Autofluorescent components in ECM of superficial and transitional layers of human articular cartilage for patient 2. The extracted normalized abundances of: (**g**) collagen I, (**h**) collagen II, (**i**) ECM (**j**) FAD, (**k**) NADH, and (**l**) RR is presented for patient 2. Red –untreated superficial (OA Sup), violet – superficial layers with treatment A (Tr A Sup), blue untreated transitional (OA Tra) and green (Tr A Tra) – transitional layers with treatment A. Additionally, for patient 2, treatment B for superficial (Tr B Sup) and transitional layer (Tr B Tra) is presented in pink and yellow respectively.

We performed statistical analysis to compare the relative amount of the extracted fluorophores in the superficial and transitional layers of the control (untreated) OA human articular cartilage and the same cartilage subjected to treatments, (Tr-A and Tr-B) as listed in Supplementary Table S1. The mean abundance of collagen I, II, ECM, NADH, FAD and the RR for patient 1 and 2 are shown in Fig. 3.

We found that the key ECM components, collagen I and II in the superficial and the transitional layers were significantly different in the OA and treated (Tr-A & Tr-B) samples (Table 2). For example, following treatments A and B, the levels of collagen I in the superficial layer increased in a statistically significant way (Patient 1+Tr-A: 1.08 vs 0.66, Patient 2+Tr-A: 1.98 vs 1.75 and Patient 2+Tr-B: 1.4 vs 1.31). The levels of collagen II in the transitional layer following treatments A and B also increased (Patient 1+Tr-A: 0.81:0.46, Patient 2+Tr-A: 1.05:0.57 and Patient 2+Tr-B: 1.35:1.26).

**Table 2:** Relative amounts and ratios of cartilage fluorophores in the superficial and transitional layers in human samples.

Extracted Fluorophores	Patient 1		Patient 2			
	Sup	Tra	Sup	Tra	Sup	Tra
	Tr-A/OA		Tr-A/OA		Tr-B/OA	
Col I	1.08	0.66	1.98	1.75	1.40	1.31
Col II	0.46	0.81	0.57	1.05	1.26	1.35
NADH	0.85	0.65	0.77	0.43	0.70	0.80
FAD	1.14	1.85	1.98	1.75	1.40	1.31
RR	1.40	2.90	2.63	4.18	2.01	1.62
ECM	0.93	1.05	0.89	1.14	0.80	0.87

We also calculated the ratios of relative amounts of fluorophores in the superficial and transitional layers (Sup/Tra) in the treated cartilage samples (Table 3) to interpret the results of treatment monitoring. As there was no data available for healthy human cartilage for different fluorophores, we compared the untreated human ratio (Sup/Tra) to a reference standard (RS) from healthy bovine cartilage. As such, the regenerative cartilage treatment was considered to have had an effect when the abundance of collagen I in the superficial layer increased (Sup/Tra > 1) and collagen II in the transitional layer decreased (Sup/Tra < 1) after treatment; with higher RR in transitional layer (Sup/Tra < 1). The outcomes of treatments applied here are indicated using the symbols (+) for successful, and (−) for unsuccessful while ≈ indicates no noticeable effect. We found that the treatment A was successful (with respect to Col-I/Col-II/RR) for both patients 1 (+/+/+) and 2 (+/+/+). The ratios (Sup/Tra) of collagen I, II and RR suggest that treatment A (+/+/+) was more successful than treatment B (+/≈/−), noting that Treatment 2 was only applied to patient 2 samples. We found minimal changes in collagen I following these treatments in patient 2 (1.13 and 1.07 for treatments A and B) compared to patient 1 (1.62 for treatment A).

**Table 3:** Ratios of relative amounts of fluorophores in superficial and transitional layers.

Extracted Fluorophores	Bovine		Patient 1		Patient 2			
	(Sup/Tra)		(Sup/Tra)		(Sup/Tra)		(Sup/Tra)	
	value	RS*	Tr-A		Tr-A		Tr-B	
Col I	2.75	>1	1.62	+	1.13	+	1.07	+
Col II	0.72	<1	0.57	+	0.55	+	0.93	≈
NADH	0.57	<1	1.30	−	1.80	−	0.87	+
FAD	0.54	<1	0.62	+	1.13	−	1.07	≈
RR	0.70	<1	0.48	+	0.63	+	1.24	−
ECM	1.53	>1	0.88	−	0.78	−	0.92	≈

*RS = reference standard; heathy bovine cartilage was used as a reference standard for a healthy ratio of given fluorophores between superficial and transitional layers. A score >1 indicated a higher level in superficial compared to transitional layer, while <1 indicated the opposite. For patients, + indicates that the ratio matched the proportions seen in the reference standard, − indicates the opposite was observed while ≈ indicated proportions were approximately equivalent.

Based on work on rabbit ear chondrocytes an RR > 1.8 has been recomended for differentiating chondrocytes originating from intact elastic cartilage from chrondocyctes originating form abnormal cartilage^40^. In this study treatment A improved the RR of the OA transitional cartilage (0.67 & 0.99 for patient 1 & 2 respectively) into this normal range for both patients (1.95 & 1.99 in Fig. 3f and l respectively), indicating changed metabolism after regenerative treatment, consistent with intensified chondrocyte metabolism^23,41,42^. The metabolism of the superficial cartilage was also improved for patients 1 and 2 (Fig. 3f and l), but not into the normal range. Similarly, treatment B in patient 2 did not clear the RR threshold defined for regenerated cartilage (1.8 vs 1.6; Fig. 3l) although improvements were seen.

## Discussion

We report here label-free, non-invasive identification of collagen I, collagen II, ECM, and the co-enzymes FAD and NADH by using hyperspectral microscopy imaging of endogenous tissue fluorescence in healthy bovine articular cartilage and human OA articular cartilage, for the first time. An additional fluorophore, UMC, which could not be identified, was also distinguishable. We were unable to identify any other collagens in the cartilage (i.e. collagen VI, collagen VIII, and collagen IX) because chemically pure reference fluorophores are required to define their spectral profile; such collagens are not commercially available. Prior to our work the only method to identify collagen I and II was by using exogenous labelling^16,26,36,43^. The other label-free imaging method, SHG imaging also used here is not sensitive to collagen type (I, II, III, V, XI)^37–39^. The ratio between forward and backward SHG signal reflects the organization of total collagen fibrils^30^. As such it only provides the total ECM signal.

Our results in healthy bovine articular cartilage indicate that levels of collagen I and collagen II differ between the superficial and transitional layers, which is congruent with previous research^43–45^. The accumulation of collagen I in the superficial layer was associated with lower levels of NADH and increased FAD (indicative of greater utilisation of oxidative phosphorylation compared to glycolysis^46^), whereas in the transitional layer the accumulation of collagen I was mostly independent of metabolic state. This suggests that alternative synthetic pathways in chondrocytes may be driving collagen synthesis between fibrocartilage types. Overall, FAD, NADH and RR were observed to be higher in the transitional layer compared to the superficial layer, indicating a less oxidised, more hypoxic state and decreased metabolic activity in the superficial layer^47,48^. This lower metabolic rate in the superficial layer is reported in the literature^49^ and related to two factors; lower mitochondrial volume and lower basal oxygen consumption. Normally, the transitional layer is intrinsically hypoxic, and cells produce their energy mainly by glycolysis^49^. Switching to oxidative phosphorylation, an aerobic energy production mechanism, may indicate a cellular response to *ex vivo* conditions in the cartilage chip. A previous study on oxygen concentration in cartilage found that it was greatly dependent on cartilage thickness^47^. This finding may therefore be a consequence of the thin slices of cartilage used in this experiment being exposed to atmospheric oxygen.

In OA human articular cartilage HS microscopy was capable of detecting treatment effects as indicated by changes in ECM composition within the superficial and transitional layers – as well as the ratios of collagen I to collagen II between the layers – pre and post treatment. Significantly, although non-OA human articular cartilage was not available for comparison, when treated human OA cartilage was compared to healthy bovine cartilage the HS characteristics observed showed that in most cases these changes resulted in the OA cartilage more closely resembling healthy cartilage. Similarly, the RR of chondrocytes in treated cartilage was improved or restored to the level seen in chondrocytes from healthy (rabbit ear) cartilage^40^. Overall, these findings support that the hyperspectral imaging of the reported features – collagen I, collagen II and RR – are sensitive to improvements in cartilage health and could be used to monitor patient progression and discern between different stages of treatment at the intrapatient level.

This study has shown that HS microscopy could be used to investigate the molecular composition of articular cartilage and non-invasively examine treatment response. It did not aim to validate the effectiveness of the experimental therapy applied here to the human OA articular cartilage. Apparent differences in treatment response observed between the samples taken from the two patients included in this study could be a consequence of the patients coming from different age groups with different levels of cartilage degradation.

Hyperspectral imaging of autofluorescence is a non-invasive technique which is suitable for long-term longitudinal monitoring of a single sample. In our hyperspectral system the tissue is only exposed to single µWs of light power over no more than 5 seconds per channel, with no observable detrimental effects on metabolism^17,50^. We envisage that in the future this technique will be available endoscopically and deployable through small incisions to collect complex molecular information on the status of cartilage. The technique may require lower magnification than 40X, or higher excitation irradiances than those used in the present work, so that short exposure time reduces motion artefacts. Additional methods will need to be introduced to deal with curved surfaces of cartilage *in vivo*. Such a methodology would be valuable for diagnostics, characterisation, and treatment planning for cartilage disease and damage.

## Supplementary information

Supplementary Material
