# Supplementary material for: Non-Invasive Monitoring of Functional State of Articular Cartilage Tissue with Label-Free Unsupervised Hyperspectral Imaging
Source: Sci Rep. 2019 Mar 13;9:4398. doi: 10.1038/s41598-019-40942-7 (PMC6416344; doi:10.1038/s41598-019-40942-7)
Supplement: Supplementary file 1 — Supplementary Material [file 41598_2019_40942_MOESM1_ESM.pdf]

Supplementary Material

Non-Invasive Monitoring of Functional State of Articular Cartilage Tissue with Label-Free Unsupervised Hyperspectral Imaging

Saabah B. Mahbub[1,2], Anna Guller[1,2], Jared M. Campbell[2], Ayad G. Anwer[1,2], Martin E. Gosnell[1,3], Graham Vesey[4], Ewa M. Goldys[2,*]

[1] ARC Centre of Excellence for Nanoscale Biophotonics, Macquarie University, NSW, Australia.

[2] Graduate School of Biomedical Engineering, UNSW Australia, Sydney, NSW 2052, Australia.

[3] Quantitative Pty Ltd, 116-118 Great Western Highway, Mt. Victoria, NSW 2786, Australia.

[4] Regeneus Pty Ltd, 25 Bridge Street, Pymble NSW 2073, Australia.

[*] **Corresponding author, E-mail address: Saabah B. Mahbub (s.mahbub@unsw.edu.au) and Ewa M. Goldys (e.goldys@unsw.edu.au)**

1 Background of articular cartilage

Hyaline cartilage is an important connective tissue covering the surface of bones. It is primarily made up of collagens which comprise about 60% of its dry weight [1] as well as distributed specialised chondrocyte cells [2] . The chondrocytes produce extracellular matrix (ECM) composed of collagen fibres, proteoglycan, and elastin fibers [3] which contribute to cartilage regeneration. Four different zones (superficial, transitional, deep and calcified) can be identified in a cross-section of articular cartilage tissue between its surface and the subchondral bone as shown in Figure S1.

The superficial layer of cartilage makes up 10% to 20% of the thickness of articular cartilage (~0.25 mm) and has the highest collagen content of all the zones [1]. The collagen fibrils are densely packed and are aligned in a highly organized manner parallel to the articular surface. The chondrocytes in this zone are elongated in shape. Figure S1 illustrates the superficial zone with the red colour area. The Transitional zone (middle zone) makes up 40% to 60% of the articular cartilage volume with a thickness 0.3 to 1 mm, and is shown with a green colour background in Figure S1. The collagen fibrils are thicker, aligned loosely and are not parallel to the surface. Chondrocytes in this layer are more rounded than the superficial zone [1]. Only the Superficial and Transitional layers will be considered in our investigation of the regenerative process of cartilage.

On the other hand, the deep zone is up to 30% of the cartilage (1 to 1.5 mm). The collagen fibrils are large in diameter and aligned perpendicular to the articular surface. This layer has the highest proportion of proteoglycans and the lowest concentration of water [1,4]. Here chondrocytes are arranged in a columnar fashion, parallel to the collagen fibres. The calcified zone lies directly on the subchondral bone and contains small cells in a chondroid matrix that has apathetic salts scattered through it [4]. Normally, the calcified zone consists of the subchondral bone and cancellous bone, which are clearly separated by tidemarks.

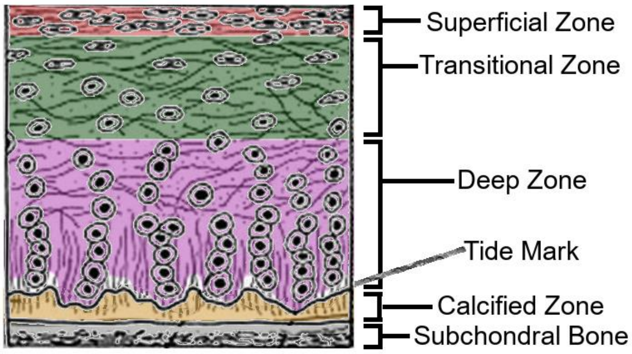

Figure S1: Schematic diagram of articular cartilage.

2. Image acquisition method

2.1 Hyperspectral Imaging

Our hyperspectral system consists of a basic fluorescence microscope (Olympus iX71™) with a 40× water U12™ series objective, with transmission in the UV range. The excitation wavelengths were centred at 334, 365, 375, 385, 395, 405, 415, 425, 435, 455, 475, 495 nm with each about 10 nm wide. In this study, three epifluorescence filter cubes were used to generate a total of 18 specific channels (table S2) to measure single photon-excited emission of biological samples. The excitation sources were coupled by an optical fibre bundle with a 5 mm fused silica hexagonal homogenizer [5]. These produced an approximately Gaussian illumination over the sample plane, whose flatness was further corrected digitally during preprocessing [6]. Hyperspectral images were captured by an EMCCD camera (Andor iXON™ 885 DU). The sensor size was 1002×1004 pixels.

For image segmentation, the area with chondrocytes and their surroundings were selected manually using a superimposed DIC image and images from channels 2 and 7. Approximately four regions were selected from each image of the cartilage (Table S1). Three images from each side of the cartilage samples were taken for each type of treatment.

2.2 Data processing method of hyperspectral imaging

A set of three hyperspectral reference images (i.e. calibration, water, and dark images) were taken using our microscope system before the beginning of each experiment. These reference images were then used for image pre-processing. Image pre-processing comprises image equalization, primary denoising by removing undetectable pixels and outliers (spikes or dips), background illumination flattening and cell segmentation [6]. The calibration fluid is a mixture of 30 µM NADH (quantum yield 0.019) and 5µM riboflavin (quantum yield 0.24), whose spectrum spans across all our spectral channels.

After manually segmenting the area of interest (intact cartilage structures with chondrocytes), the pixel identifiers (image number, pixel coordinates, spectral channel etc.) were separately retained for indexing the layers of cartilage chips undergoing different treatments.

Data analysis was based on a linear mixing model (LMM) which postulates that the fluorescent signal at each pixel is a linear combination of a small number of component spectra called "endmembers" with respective weights corresponding to concentrations of the molecules responsible for these component spectra. These concentrations were expressed as abundance fractions [7-9]. The Robust Dependent Component Analysis [6,10] (RoDECA) algorithm was used for unmixing the dominant native fluorophores and their corresponding abundance (Section 5).

2.3 Second harmonic generation (SHG) imaging

Frozen sections (10 µM) from bovine cartilage were imaged with an inverted confocal laser scanning microscope (TCS MP SP5 system from Leica) equipped with a Spectra-Physics Mai Tai Ti: Sapphire femtosecond laser. The laser was tuned to 880 nm and the images were focused on chondrocytes. The resulting SHG signal was detected at 440 nm found to give the best quality image. The forward-scattered signal was detected and back-scattered excitation light was removed using a 680-nm short-pass dichroic mirror. Z-stacks of each frozen section were generated to describe the total collagen structure.

Both forward and backward SHG images were measured, and their analysis was carried out with Matlab™ 2016b. To avoid edge effects (i.e. the attenuation of the SHG signal at the top and bottom of the section), only the internal 8 images of each z-stack were included in the measurements. Prior to the quantification of image intensities (normalized relative amount), the images were filtered to reduce shot noise, and a minimum intensity threshold was set interactively to exclude the spaces between collagen fibres. Mean grey value limited to a threshold of each image was calculated. Total SHG intensity for each z-slice was taken as the sum of the mean intensities of the forward and backward channels.

3. Investigated samples

In this experiment, both bovine and human samples have been used. Bovine cartilage fragments were harvested from the knee joints of commercially available cattle carcasses, whereas, human knee joint samples from two patients with

osteoarthritis (OA) who were undergoing total knee replacement surgeries were collected fresh, within 1 hour after the operation. (details are given in Section 2.2 and 2.3).

Supplementary Table S1: Sample preparation set from bovine and human

Sample Set	Source	Cartilage Layer	Functional state of the tissue	Treatment applied	No of image	No of areas	Section Reference
1	Bovine 1	Superficial	Intact	No	3	4	Section 3.1 & Fig 1 (a-g) Fig 2 (a-p)
2		Transitional	Intact	No	3	5	
3	Bovine 2	Superficial	Intact	No	2	5	
4		Transitional	Intact	No	2	4	
5	Bovine 3	Superficial	Intact	No	2	4	
6		Transitional	Intact	No	2	4	
7	Bovine 1	Superficial	Intact	No	whole	10 layers	Section 3.1 & Fig 1 (h-j)
8		Transitional	Intact	No	whole	10 layers	
9	Human 1	Superficial	OA	No / OA	3	5	Section 3.3 & Fig 3 (a-f)
10		Transitional	OA		3	5	
11		Superficial	OA		3	4	
12		Transitional	OA		3	4	
13		Superficial	OA	Yes / Treatment A	3	5	
14		Transitional	OA		3	5	
15		Superficial	OA		3	4	
16		Transitional	OA		3	4	
17	Human 2	Superficial	OA	No / OA	3	4	Section 3.3 & Fig 3 (g-l)
18		Transitional	OA		3	4	
19		Superficial	OA		3	3	
20		Transitional	OA		3	3	
21		Superficial	OA	Yes / Treatment A	3	4	
22		Transitional	OA		3	4	
23		Superficial	OA		3	3	
24		Transitional	OA		3	4	
25		Superficial	OA	Yes / Treatment B	3	4	
26		Transitional	OA		3	3	
27		Superficial	OA		3	3	
28		Transitional	OA		3	4	

4. Context of hyperspectral unmixing

In hyperspectral imaging, 2-D (two-dimensional) images (N pixels each; here $N \approx 10^6$) of the same sample are taken in L different spectral channels (L=18 in our study). The recorded fluorescence signals from the hyperspectral dataset described by a matrix $\boldsymbol{y} = [\, y_{ki} \,]$, where y_{ki} is the pixel value in channel k, (k=1,..,L) of image pixel i, (i=1,...,N) [7]. This matrix can be considered as a sum of the noiseless signal matrix $\boldsymbol{x}$ and the matrix of image noise $\boldsymbol{n}$ where $\boldsymbol{y} = \boldsymbol{x} + \boldsymbol{n}$. Further analysis is based on a linear mixing model (LMM) which postulates that the observed spectrum in each

pixel is a linear combination of a small number p of component spectra called "endmembers" with respective weights (that is a concentration of the spectrum component) called "abundance" fractions [7-9]. For the unmixing to be accurate, the number of components needs to be smaller than the number of channels ($p<L$) Algebraically, in the LMM, the noiseless signals $\boldsymbol{x}$ are expressed with the aid of an endmember matrix $\boldsymbol{M} = [M_{kj}]$, (where, k=1,...,L, j=1,...,p) is weighted with abundance fractions specified in the abundance matrix $\boldsymbol{s} = [s_{ji}]$, ($j$=1,...,$p$, i=1,...,N) according to

$$\boldsymbol{x} = [x_{ki}] = \sum_{j=1}^{p} M_{kj} s_{ji} = \boldsymbol{Ms}. \qquad (1)$$

Due to physical constraints, the abundance fractions in each pixel must be positive ($s_{ji} \geq 0$) and they must add to unity $\sum_{j=1}^{p} s_{ji} = 1$ [11,12]. The abundance matrix $\boldsymbol{s}$ contains the abundance fractions of each endmember for all N pixels and this makes it possible to easily construct the abundance map for the imaged sample. The L-dimensional vectors $\overrightarrow{y_i}$ forming columns of the matrix $\boldsymbol{y}$, represent the pixel spectra, while the L-dimensional vectors $\overrightarrow{M_j}$ forming columns of matrix $\boldsymbol{M}$ represent the endmember spectra. Equation (1) can be expressed as

$$\overrightarrow{y_i} = \overrightarrow{M_1} s_{1i} + \cdots + \overrightarrow{M_p}\, s_{pi} \qquad (2)$$

This formulation makes it possible to see that the Equation (2) describes a convex set in L-dimensions span by the vectors $\overrightarrow{M_j}$. Therefore the pixel spectral vectors from the matrix $\boldsymbol{x}$ form a convex hull (a simplex) whose vertices are determined by the endmember spectral vectors [13]. There is p such vectors, so the convex hull has p vertices. The presence of noise $\boldsymbol{n}$ creates a fuzzy boundary of this convex hull.

In the unsupervised unmixing approach both matrices $\boldsymbol{M}$ and $\boldsymbol{s}$ are unknown, and, additionally, we do not know the number of endmembers p . As previously indicated, the observed pixel signals in the hyperspectral dataset $\boldsymbol{y}$ are affected by the sensor noise coming from the image sensor and there may be other experimental sources of errors which are impossible to eliminate. Equation (2) then becomes:

$$\boldsymbol{y} = \boldsymbol{Ms} + \boldsymbol{n}, \qquad (3)$$

The noise matrix $\boldsymbol{n}$ is also unknown. The procedure of unsupervised unmixing aims to establish the number of component spectra of a linear mixture and their identity, specifically to estimate optimal p, $\boldsymbol{M}$ and $\boldsymbol{s}$ none of which are known *a-priori*. Such statistical optimisation can give accurate results because hyperspectral imaging generates large datasets, so a large number N of experimental pixel spectra are available for analysis.

5 Hyperspectral Unmixing with RoDECA procedure

In this work, the Robust Dependent Component Analysis [6,10] (RoDECA) algorithm is used for extracting the native fluorophores and their corresponding abundance. RoDECA is an unsupervised hyperspectral unmixing method and well suited to highly mixed datasets, where there is no pure pixels present which is apparent for biological samples. In the RoDECA analysis, the intermediate endmember matrix is initially estimated blindly [6], by simulating the abundance matrix as a mixture of Dirichlet densities [12]. The Dirichlet densities approach automatically enforces the constraints of non-negativity and unity sum, and thus it maintains the physiological constraints of the presence of native fluorophores. The soft negative function of RoDECA analysis gives more robust extracting of fluorophores and introducing of spectral normalization ensures the non-negativity and normalized intensity values at each channel for each extracted fluorophores' spectra.

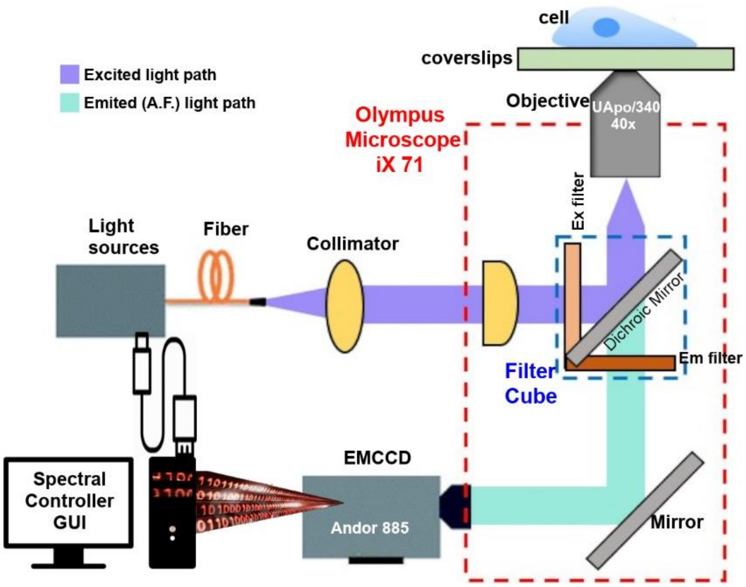

Figure S2: Schematic diagram of Hyperspectral hardware setup with optical path of Olympus iX71™ microscope (marked with red dotted line) where filter cube is spotted with blue dotted line

6 Hyperspectral imaging parameters

At first, the culture medium with DMEM (Dulbecco's modified eagle's medium, Sigma-Aldrich, D5796) medium containing 10% fetal bovine serum (FbS; Gibco, Catalog No: 16000-044) and the treatment solution was removed (as it produces undesirable background fluorescence). After that, cartilage chips were washed with PBS three times. Then 1 ml of non-fluorescent Hanks Balanced Salt Solution (HBSS) was added to each dish for imaging. The cartilage chips were imaged with a 35 mm plastic culture dishes with 18 mm well and 1.5 gridded coverslip bottoms (Cell E&G, USA, and GDB0004-200). During the imaging session, the temperature was controlled at 37° C. The layer located over a reference grid at the bottom of the dishes were imaged using 18 fluorescence channels. These gridded coverslips are also useful for further correlation of hyperspectral imaging with other microscopy modalities. A single wavelength image was typically acquired for from 0.5 to 15 seconds. Depending on the sample quality and spectral response, different image averaging has been used and approximately 3-4 minutes is needed for the entire stack of images from 18 spectral channels.

Table S2: Hyperspectral Imaging parameter for human cartilage sample

Channel	Excitation (nm)	Emission (nm)	Filter cube	EM gain	Exposure (sec)	Average	Power (µW)
1	334	450±30	1	80	2.5	4	0.046
2	365±5	450±30	1	1	0.012	4	6.50
3	375±5	450±30	1	1	0.012	5	2.83
4	334±5	585±17	2	299	4.99	5	0.020
5	365±5	585±17	2	4	0.05	4	6.40
6	375±5	585±17	2	1	0.02	5	10.51
7	385±5	585±17	2	1	0.02	4	17.77
8	395±5	585±17	2	1	0.02	4	13.33
9	405±5	585±17	2	2	0.03	4	9.39
10	415±5	585±17	2	1	0.02	4	22.40
11	425±5	585±17	2	1	0.02	4	20.90
12	435±5	585±17	2	2	0.02	4	27.50
13	455±5	585±17	2	2	0.07	3	14.80

14	470±5	585±17	2	2	0.02	3	42.80
15	495±5	585±17	2	10	0.08	3	12.01
16	405±5	700 long pass	3	8	0.1	3	9.50
17	455±5	700 long pass	3	15	0.1	3	15.09
18	495±5	700 long pass	3	10	0.1	3	9.97
DIC	white light	585±17	2	1	0.005	2	-

7 Hyperspectral imaging parameters of thick tissue (cartilage chip)

During the imaging session, images of both sides of cartilage were taken to make a comparison between the different layers of cartilage (superficial, transitional). Cartilage chips have a relatively broad cross-section (i.e. thickness) of around 500-1000 µm compared to a typical value of 10-20 µm for plated cells, where z-stack imaging is not necessary. Moreover, there are lots of scattering problem for cartilage chips, because of cartilage contains 60-80% water and leads the problem of improper visualization and focusing of the chondrocytes into opaque cartilage tissue. Typically, scattering problems are solved by using optical clearing via immersion in iohexol [14]. However, we found that iohexol significantly induced ROS which alters cell/tissue fluorescence [15] and would have confounded the experiment. To solve these problems, we developed some modifications, with respect to both hardware and software.

7.1 Hardware modifications for accurate focusing

Firstly, a half mirror was using to control the focus of the light on the sample. This was a very effective methodology for focusing in terms of the alternating the optics. The 50:50 mirror (BSW10R 25×36 mm 50:50 UVFS plate beam splitter, LASTEK Pty. Ltd.) can transmit 50% of the light and the other 50% of reflected light can coincide the structure inside the cartilage tissue. This beam splitter was installed inside the filter-cube of the inverted microscope. This specific filter cube was used during only during the scanning and focusing period. Secondly, in this study, a white light source (coupled with 100 W mercury lamp) was installed through the optical fibre from homogenizer, without hampering the original arrangement HS light sources. Both white source and the half mirror made the focusing easier than the single light source.

As cartilage chips are placed into the petri dishes for imaging there is an uneven gap between the samples and the glass coverslip (Figure S3). For that reason, we could not use the Petri dish's grid for the reference plane for focusing. Therefore, we put the sample on top of a glass bottom dish with 1 ml HBSS solution. Then we put another glass slide on top of it and took out the rest amount of the solution carefully with a pipette. The capillary effect between the glass slide (both side) and the Hanks solution, squeezed the cartilage chip, and that caused the cartilage chip to be flattened easily, allowing the use of the reference grid for further focussing.

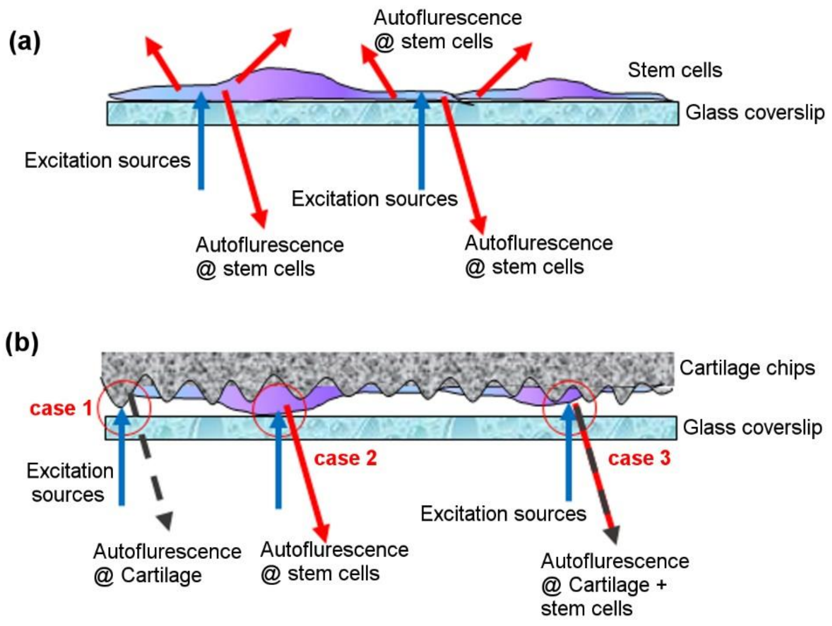

Figure S3: Schematic diagram of HS imaging of (a) a standard procedure for stem cell on a glass coverslip, (b) during stem cell over cartilage chip.

7.2 Sample modifications: formation of grids on cartilage

In this experiment, grids were produced on the smooth cartilage side using picosecond laser (Laser type: SuperrapidHE, Model: 3D MicroMac micro structC from ANFF, OptoFab Node, Macquarie University). These serve two purposes. Firstly, an etched grid makes it possible to generate controlled defects in specified locations by laser ablation of the cartilage surface (up to transitional layer), presented in Figure S4. Secondly, this grid is used for referencing in other imaging modalities, as for an example for confocal microscopy after autofluorescence imaging (Figure S5). A picosecond laser (SuperrapidHE, wavelength: 266nm-4th HarmonicNd, Pulse duration: 8 ps and Spot size: 12 µm) was used for etching the grid on the cartilage. Due to the softness of the cartilage, very low power (50 mW with pulse repetition frequency-100kHz and scan speed-100mm/s) was used for this etching work, which allows to etch the structure with the fluence of 0.44 J/cm^2.

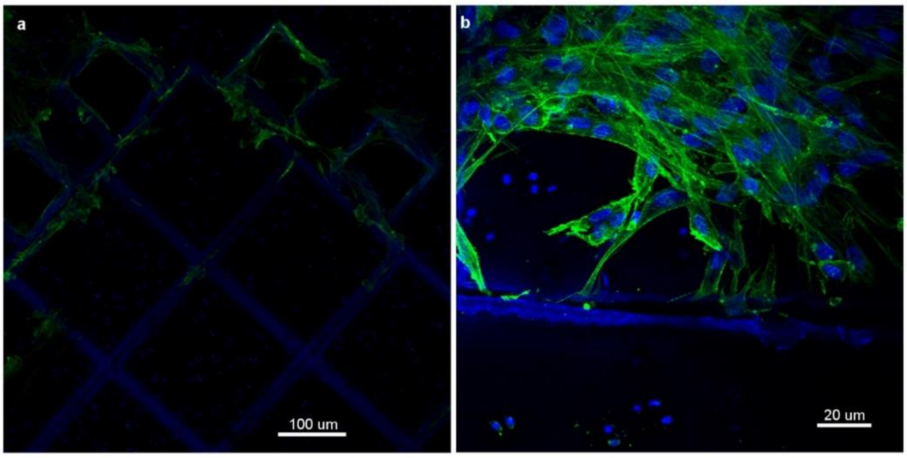

Figure S4: MSC Stem cells over smooth side of non-fixed cartilage (a) 20x with confocal image after staining. (b) 10x with MSCs cell attachment over the etched grids (defective portion) on smooth side of the cartilage.

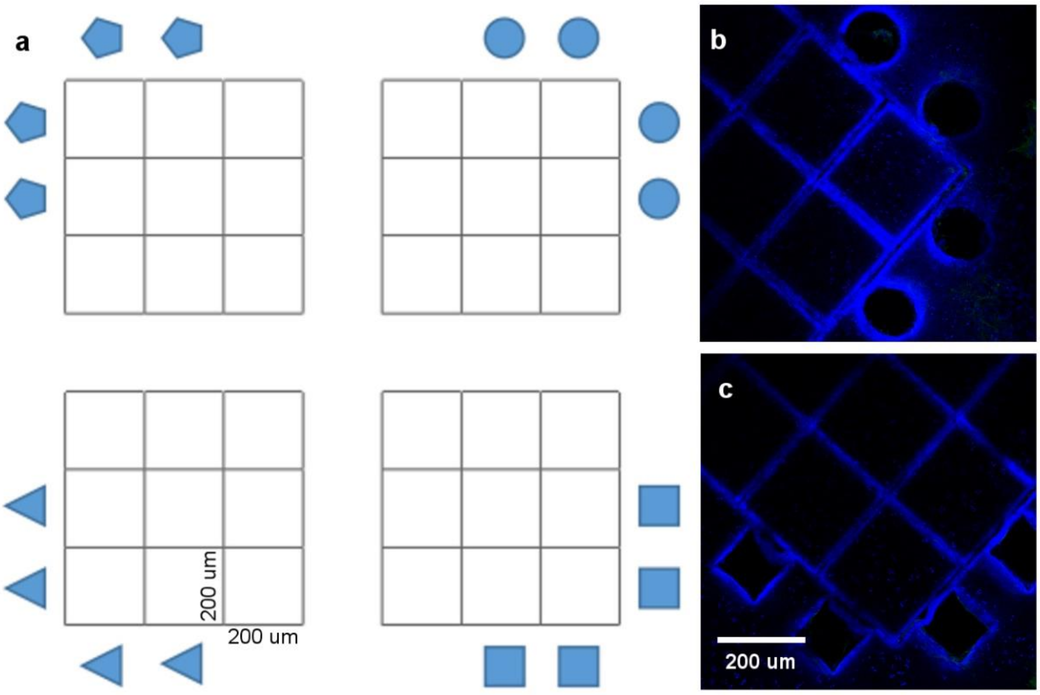

Figure S5: (a) Grid design for cartilage, (b) grids with 'round shape' are taken with confocal microscopy with Hoechst 33342™, (c) grids with 'square shape' are taken with confocal microscopy with Hoechst 33342™.

8 Reference excitation-emission matrix (EEM) for different types of collagen

The main aim of this study was to monitor the regeneration process of the cartilage tissue in terms of autofluorescence. To facilitate this, RoDECA was used to analyse the HS data from cartilage chips. This unmixing of the native fluorophores could help us to unveil the biochemical information of the chondrocytes and the extracellular matrix. But it is important to crosscheck the extracted spectra with the reference excitation-emission matrix (EEM), which can cover all the used spectral channels. But unfortunately, there is no comprehensive EEM data for different types of collagen (I, II, III, IV, V etc.) in aqueous solution. Some studies have reported the EEM of collagen I and II [16] in terms of solid fibres, which could not find any significant peak difference. However, other studies did not differentiate among the collagen types for their tissue experiments [17-19]. That is why it is necessary to have a complete EEM of the collagen (I, II, III, V) at their purest state to facilitate the unmixing analysis.

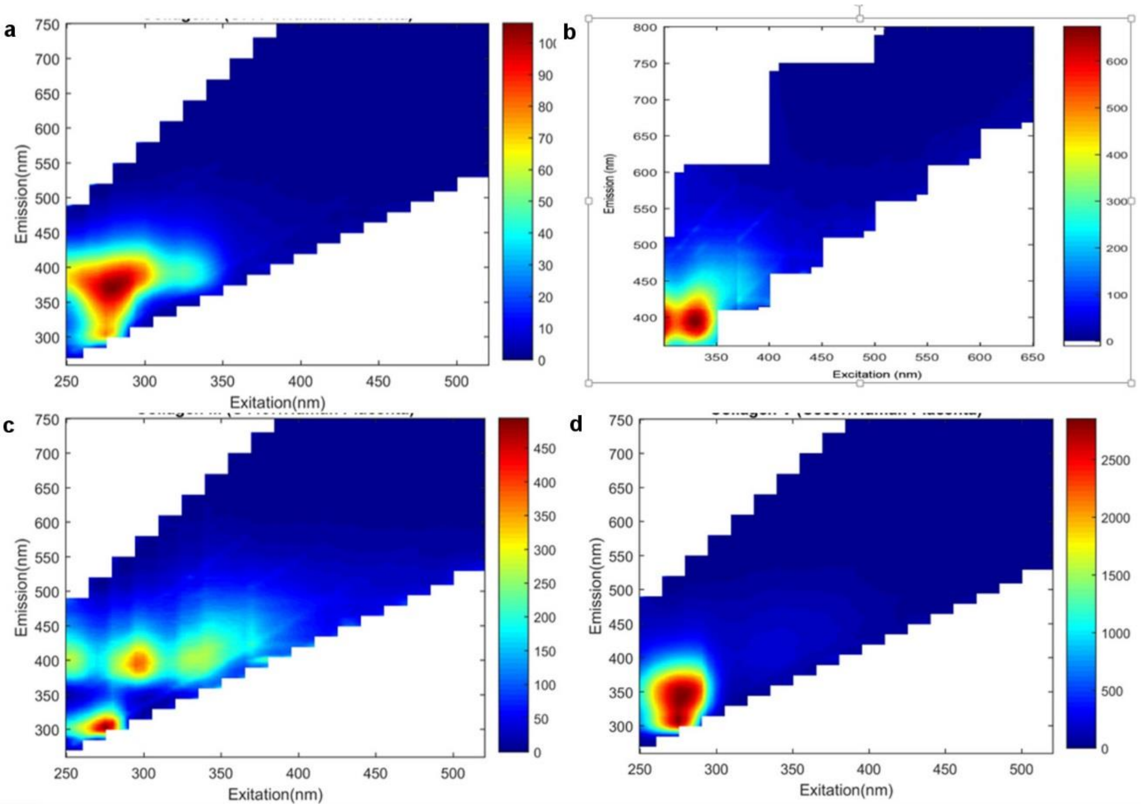

Figure S6: EEM for (a) collagen I, (b) collagen II, (c) collagen III and (d) collagen V in aqueous solution.

Although different types of collagen exist, they are all composed of molecules containing three polypeptide chains arranged in a triple helical conformation. Slight differences in the primary structure (amino acid sequence) establish differences between the types. Collagen I from human placenta (Product Number C7774, CAS Number: 9007-34-5, from Sigma) was used for this experiment and shown in Figure S6 (a). This collagen is Bornstein and Traub Type I. Type I collagen is a component of skin, bone, tendon, and other fibrous connective tissues. Type I collagen differs from other collagens by their low lysine hydroxylation and low carbohydrate composition [20,21]. This product is an acid soluble collagen. It was dissolved in water with acetic acid added to pH 3.0 with (2.5 mg/ml), yielding an opalescent, colourless solution. An entire EEM was taken at excitation from 250 nm to 650 nm and emission from 270 nm to 750 nm with Cary Eclipse.

Collagen II from bovine tracheal cartilage was used (Product Number C1188, CAS Number:9007-34-5, from Sigma) was represented in Figure S6 (b) which was dissolved in water with acetic acid added to pH 3.0 with (2.5 mg/ml), yielding an opalescent, colourless solution. Collagen III (C4407/Human Placenta from Sigma) and collagen V (C3657/Human Placenta from Sigma) were liquefied in water with acetic acid added to pH 3.0 with (2.5 mg/ml) and illustrated in Figure S6 (c) and (d) respectively.

9 Simplex Identification of the examined samples

9.1 Simplex presentation of the bovine samples

RoDECA analysis was used for unmixing the hyperspectral dataset for this study. With RoDECA analysis, five fluorophores have been successfully identified with using the maximum Dirichlet mode of 5. From where we can have ascertained the four fluorophores with the reference bank (Supplementary Section 5). The Collagen I, Collagen II, FAD, and Free NADH have been found with soft vertices in the projection onto the first and second subspace vectors in the Figure S7 (a). Blue colour dots represent the transitional layer and red coloured dots represents the superficial layer. Reference spectra (black, from Supplementary Section 5) and extracted spectra (in red, from RoDECA unmixing) is presented in Figure S7 (b) collagen I, (c) collagen II, (d) Unknown ECM component, (e) FAD and (f) NADH. Furthermore, Figure S7 (g, h) represents the DIC image of intact bovine articular cartilage (sample set 1 for superficial and sample set 2 for transitional layer, as listed in Table 1 and Figure 2 in the main article).

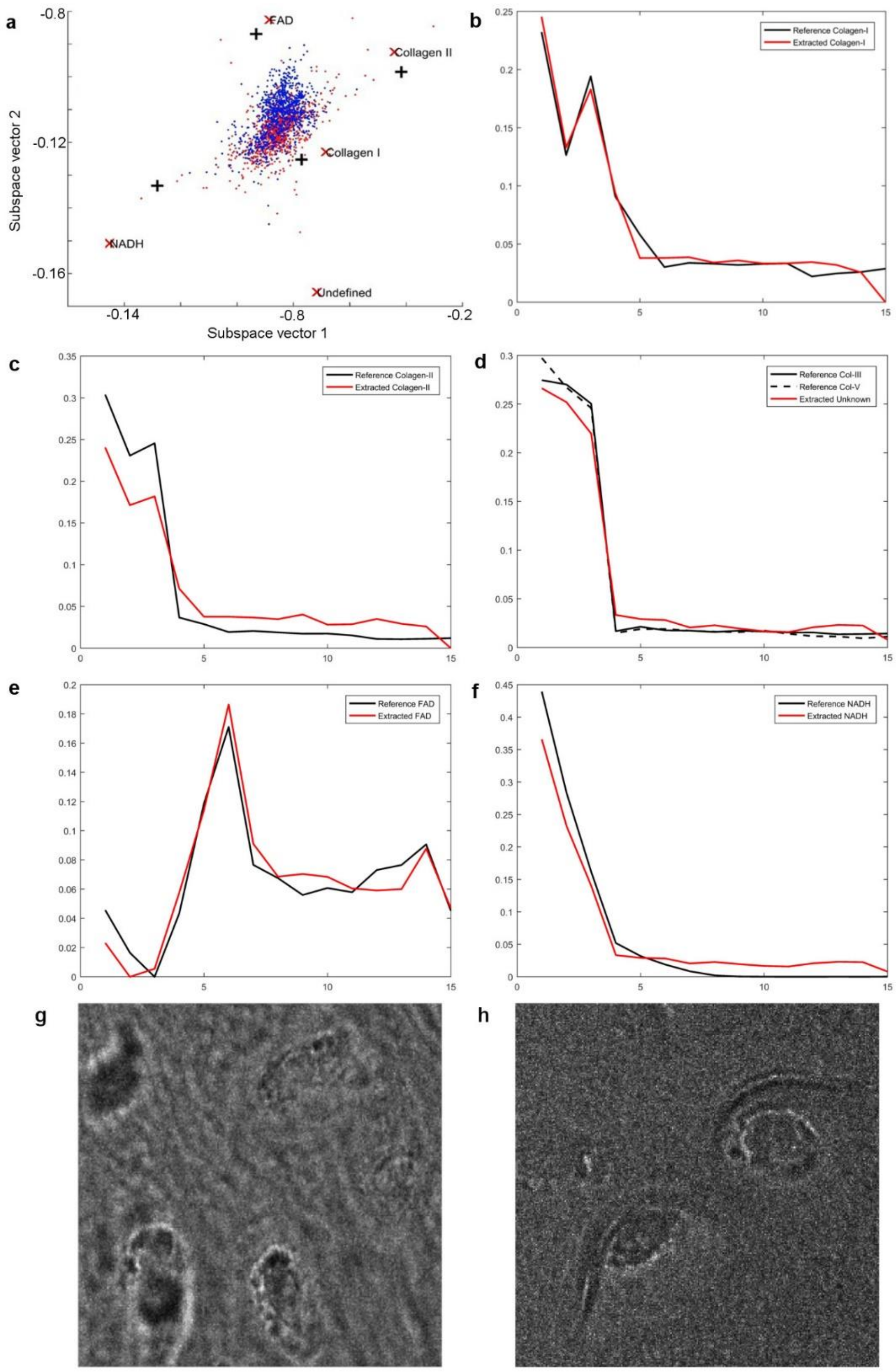

Figure S7: (a) Simplex representation of superficial and transitional layer of bovine cartilage with extracted fluorophores with respect to subspace vector 1 and subspace vector 2. Blue colour dots represent the transitional layer and red coloured dots represents the superficial layer. (b-f) Reference spectra (black) and extracted spectra (in red) representation of (b) collagen I, (c) collagen II, (d) Unknown ECM component, (e) FAD and (f) NADH. (g, h) DIC image of intact bovine articular cartilage (sample set 1 for superficial and sample set 2 for transitional layer, as listed in Table 1 and Figure 2 in the main article)

9.2 Simplex presentation of the human samples

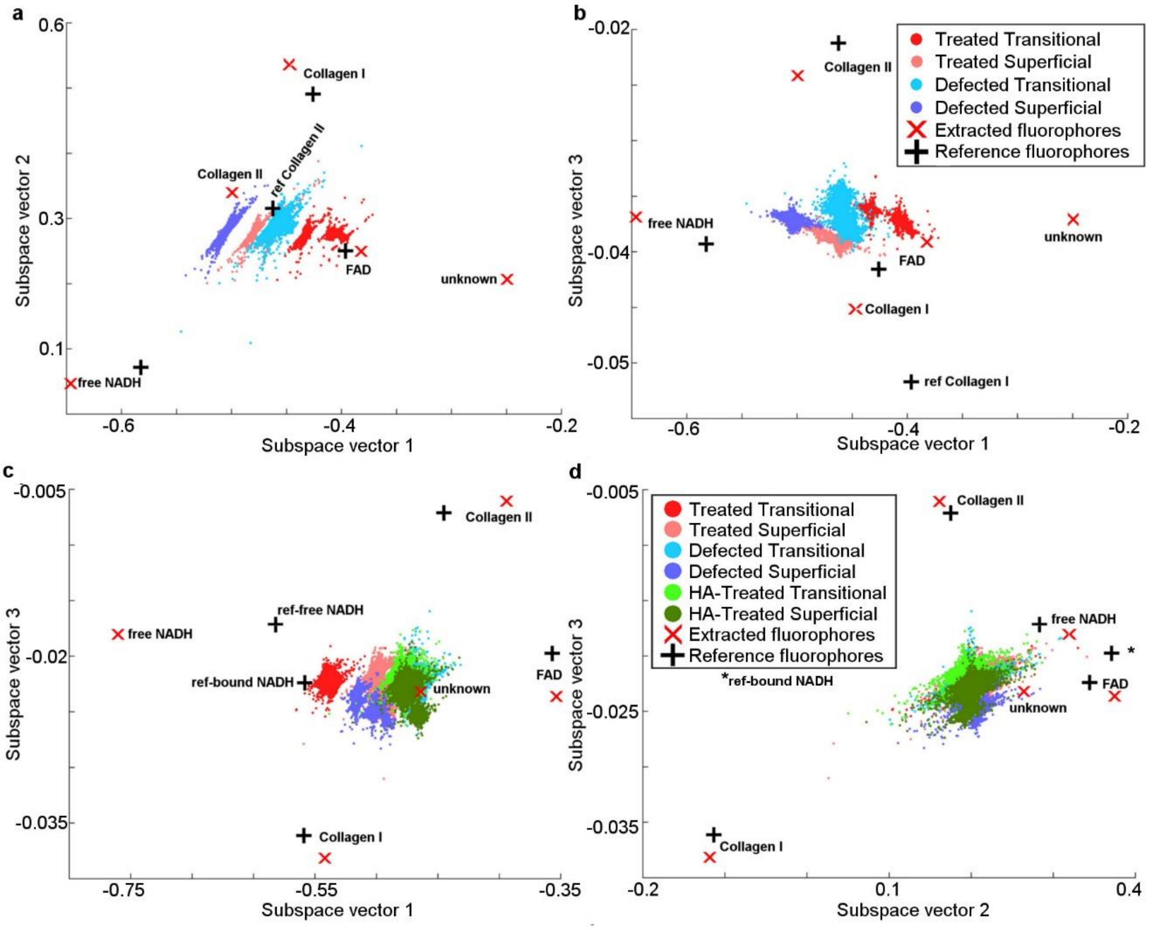

Figure S8: (a, b) Simplex representation of defective and treated human cartilage chips with extracted fluorophores with respect to (a) subspace vector 1 and subspace vector 2 and (b) subspace vector 1 and subspace vector 3. (c, d) Simplex representation of defective, cytokine-treated, and HA-treated human cartilage chips with extracted fluorophores with respect to (a) subspace vector 1 and subspace vector 3, (b) subspace vector 2 and subspace vector 3.

10 Correlation of bovine cartilages with ECM and cellular fluorophores

The ECM composition of the cartilage was statistically significantly associated with the concentrations of NADH, FAD and with the optical redox ratio, i.e. with the metabolic state of the cells. The accumulation (increased production) of collagen type I by the cells was associated with decreased accumulation of collagen type II and UMC in the tissue (especially notable in the transitional layer). In the superficial layer accumulation of collagen I was associated with the depletion of the cells' NADH, with an increase in FAD and RR. In the transitional layer, accumulation of collagen I was almost independent of the changes in cellular metabolic state. This could be attributable to the existence of alternative synthetic phenotypes in chondrocytes between the fibrocartilage types.

In the superficial layers HS abundance of collagen I correlated negatively (weak correlation) with the abundance of collagen II (R_s=-0.326; $p<0.001$); the abundance of UMC (R_s=-0.386, $p<0.001$); and abundance of NADH (R_s=-0.376, $p<0.001$); and correlated positively (weak correlation) with the abundance of FAD (R_s=0.412, $p<0.001$) and (medium correlation) with RR (R_s=0.618, $p<0.001$).This indicates that the accumulation of collagen I in the superficial layer is associated with the activation of oxidative phosphorylation and down-regulation of glycolytic pathway of energy production. This process was associated with a decrease in the abundance of collagen II and UMC. In a similar way the abundance of collagen II correlated negatively (weak correlation) with the abundance of UMC (R_s=-0.282, $p<0.001$), abundance of NADH (R_s=-0.382, $p<0.001$), FAD (R_s=-0.311, $p<0.001$) and with the RR (R_s=-0.225, $p<0.001$). This indicates that the link between the accumulation of collagen II and the metabolic state of the cells is comparatively very weak and, most probably, mediated by some other processes. However, generally, a glycolytic status favoured to accumulation of collagen II in superficial cartilage.

Table S3: Correlation between ECM fluorophores and cellular fluorophores of bovine cartilages

SUPERFICIAL LAYER -INTACT BOVINE CARTILAGE

	Collagen Type I	Collagen Type II	Unknown ECM Component	ECM, mixed signal	% of Collagen Type I in ECM	% of Collagen Type II in ECM	% of UMC in ECM	NADH	FAD	Optical Redox Ratio
Collagen Type I	1.000	-.326**	-.387**	-.027**	.894**	-.298**	-.429**	-.376**	.412**	.618**
Collagen Type II	-.326**	1.000	-.282**	.385**	-.468**	.938**	-.394**	-.382**	-.311**	-.225**
Unknown ECM Component	-.387**	-.282**	1.000	.522**	-.564**	-.510**	.975**	-.036**	-.702**	-.535**
ECM, mixed signal	-.027**	.385**	.522**	1.000	-.413**	.102**	.349**	-.671**	-.806**	-.354**
% of Collagen Type I in ECM	.894**	-.468**	-.564**	-.413**	1.000	-.311**	-.539**	-.087**	.712**	.748**
% of Collagen Type II in ECM	-.298**	.938**	-.510**	.102**	-.311**	1.000	-.587**	-.245**	-.029**	-.036**
% of UMC in ECM	-.429**	-.394**	.975**	.349**	-.539**	-.587**	1.000	.136**	-.594**	-.536**
NADH	-.376**	-.382**	-.036**	-.671**	-.087**	-.245**	.136**	1.000	.190**	-.310**
FAD	.412**	-.311**	-.702**	-.806**	.712**	-.029**	-.594**	.190**	1.000	.792**
Optical Redox Ratio	.618**	-.225**	-.535**	-.354**	.748**	-.036**	-.536**	-.310**	.792**	1.000
**TRANSITIONAL LAYER - INTACT BOVINE CARTILAGE**										
	Collagen Type I	Collagen Type II	Unknown ECM Component	ECM, mixed signal	% of Collagen Type I in ECM	% of Collagen Type II in ECM	% of UMC in ECM	NADH	FAD	Optical Redox Ratio
Collagen Type I	1.000	-.843**	-.895**	-.823**	.993**	-.757**	-.792**	.035**	.127**	-.034**
Collagen Type II	-.843**	1.000	.674**	.799**	-.866**	.959**	.449**	.036**	-.206**	-.038**
Unknown ECM Component	-.895**	.674**	1.000	.895**	-.908**	.517**	.925**	-.158**	0.001	.198**
ECM, mixed signal	-.823**	.799**	.895**	1.000	-.868**	.646**	.701**	-.121**	-.066**	.156**
% of Collagen Type I in ECM	.993**	-.866**	-.908**	-.868**	1.000	-.770**	-.776**	.023**	.149**	-.030**
% of Collagen Type II in ECM	-.757**	.959**	.517**	.646**	-.770**	1.000	.289**	.122**	-.278**	-.150**
% of UMC in ECM	-.792**	.449**	.925**	.701**	-.776**	.289**	1.000	-.221**	.130**	.273**
NADH	.035**	.036**	-.158**	-.121**	.023**	.122**	-.221**	1.000	-.957**	-.898**
FAD	.127**	-.206**	0.001	-.066**	.149**	-.278**	.130**	-.957**	1.000	.913**
Optical Redox Ratio	-.034**	-.038**	.198**	.156**	-.030**	-.150**	.273**	-.898**	.913**	1.000
** Correlation is significant at the 0.01 level (2-tailed test).										

On the other hand, for transitional layers, the abundance of collagen I correlated negatively (strong correlation) with the abundance of collagen II (Rs=-0.843; p<0.001) as well as the abundance of UMC (Rs=-0.895,

p<0.001). Its correlation with the abundance of cellular fluorophores, NADH and FAD was positive, but very weak (Rs=0.350, p<0.001 for NADH) and (Rs=0.127, p<0.001; for FAD). The correlation with RR was very weak and negative (Rs=-0.034, p<0.001), which was opposite in direction and lesser in strength compared to the observation in the superficial layer. This indicates that the accumulation of collagen I in the transitional layer is, most probably, not associated with a change in the metabolic state of the cells, but with the intensity of the collagen I production or degradation. The strong negative correlation between the abundance of collagen I, and collagen II / UMC testifies in favour of the replacement of one component by the other in the composition of ECM (i.e. increased collagen I occurs on the background of decreased collagen II and UMC). Moreover, the abundance of collagen type II correlated positively, with moderate strength (opposite to the superficial layer), with the abundance of UMC (Rs=0.674, p<0.001); weakly positive with abundance of NADH (Rs=0.036, p<0.001), and weakly negative with FAD (Rs=-0.206, p<0.001) and with the RR (Rs=-0.038, p<0.001). This indicates that the accumulation of collagen type II most probably occurs in parallel with the accumulation of UMC, resulting in hyaline type of cartilage. The link between the accumulation of collagen II and the metabolic state of the cells in the transitional cartilage is very weak and, most probably, mediated by some other processes. Here the observed negative correlations with all cellular fluorophores can be explained by the higher local concentration of collagen type II outside the cells (so, the less cells, the less NADH and FAD, and more UMC). There might be a distance-from-cell dependent UMC concentration.

Mixed HS signal of ECM in superficial layers strongly negatively correlated with NADH (Rs=-0.671; p<0.001) and FAD (Rs=-0.806, p<0.001) and weakly negatively with RR (Rs=-0.354, p<0.001). This may be attributed to the activation of ECM degradation or to an increase in cell density parallel with the change of metabolic state of the cells. In particular, the strongest observed negative correlation was between ECM and FAD (Rs=-0.806). This was an almost linear dependence, showing that if a cell shifts towards oxidative phosphorylation the overall ECM abundance decreases.

However, the mixed HS signal of ECM in transitional layers was very weakly negatively (opposite to the superficial layer) correlated with NADH (Rs=-0.121; p<0.001) and FAD (Rs=-0.066, p<0.001) and weakly positively correlated with RR (Rs=0.156, p<0.001). This indicates that the ECM abundance in the transitional layer is almost independent of cellular metabolic state.

Supplementary References:

1 Sophia Fox, A. J., Bedi, A. & Rodeo, S. A. The basic science of articular cartilage: structure, composition, and function. *Sports health* **1**, 461-468 (2009).

2 Sobol, E. N., Shekhter, A. B., Guller, A., Baum, O. I. & Baskov, A. V. Laser-induced regeneration of cartilage. *Journal of Biomedical Optics* **16**, 080902 (2011).

3 Frantz, C., Stewart, K. M. & Weaver, V. M. The extracellular matrix at a glance. *J Cell Sci* **123**, 4195-4200 (2010).

4 Scott, W. N. *Insall & Scott Surgery of the Knee E-Book.* (Elsevier Health Sciences, 2011).

5 Gosnell, M. E. *Unlocking the potential of spectral imaging for the characterization of cell and stem cell populations* Ph.D. thesis, Macquarie University, (2014).

6 Mahbub, S. B., Plöschner, M., Gosnell, M. E., Anwer, A. G. & Goldys, E. M. Statistically strong label-free quantitative identification of native fluorophores in a biological sample. *Scientific reports* **7**, 15792 (2017).

7 Keshava, N. & Mustard, J. F. Spectral unmixing. *Signal Processing Magazine, IEEE* **19**, 44-57, doi:10.1109/79.974727 (2002).

8 Keshava, N., Kerekes, J. P., Manolakis, D. G. & Shaw, G. A. 42-63.

9 Keshava, N. A survey of spectral unmixing algorithms. *Lincoln Laboratory Journal* **14**, 55-78 (2003).

10 Mahbub, S. B. *Unsupervised hyperspectral unmixing analysis for label-free quantitative identification of native fluorophores in a biological sample by a Robust Dependent Component Analysis (RoDECA)* PhD thesis, Macquarie University, (2017).

11 Manolakis, D., Siracusa, C. & Shaw, G. Hyperspectral subpixel target detection using the linear mixing model. *Geoscience and Remote Sensing, IEEE Transactions on* **39**, 1392-1409, doi:10.1109/36.934072 (2001).

12 Nascimento, J. M. P. & Bioucas-Dias, J. M. Hyperspectral Unmixing Based on Mixtures of Dirichlet Components. *Geoscience and Remote Sensing, IEEE Transactions on* **50**, 863-878, doi:10.1109/TGRS.2011.2163941 (2012).
13 Nascimento, J. M. & Bioucas-Dias, J. M. in *Geoscience and Remote Sensing Symposium, 2007. IGARSS 2007. IEEE International.* 3225-3228 (IEEE).
14 Bykov, A. *et al.* Imaging of subchondral bone by optical coherence tomography upon optical clearing of articular cartilage. *Journal of Biophotonics* **9**, 270-275, doi:doi:10.1002/jbio.201500130 (2016).
15 Jeong, B. Y. *et al.* Oxidative stress caused by activation of NADPH oxidase 4 promotes contrast-induced acute kidney injury. *PLOS ONE* **13**, e0191034, doi:10.1371/journal.pone.0191034 (2018).
16 DaCosta, R. S., Andersson, H. & Wilson, B. C. Molecular fluorescence excitation–emission matrices relevant to tissue spectroscopy. *Photochemistry and photobiology* **78**, 384-392 (2003).
17 Georgakoudi, I. *et al.* NAD (P) H and collagen as in vivo quantitative fluorescent biomarkers of epithelial precancerous changes. *Cancer research* **62**, 682-687 (2002).
18 Lewis, W., Padilla-Martinez, J. P., Ortega-Martinez, A. & Franco, W. Changes in endogenous UV fluorescence and biomechanical stiffness of bovine articular cartilage after collagenase digestion are strongly correlated. *Journal of biophotonics* **10**, 1018-1025 (2017).
19 Yu, Y. *et al.* Imaging-guided two-photon excitation-emission-matrix measurements of human skin tissues. *Journal of Biomedical Optics* **17**, 077004 (2012).
20 Bornstein, P. & Sage, H. Structurally distinct collagen types. *Annual review of biochemistry* **49**, 957-1003 (1980).
21 Andersson, H., Baechi, T., Hoechl, M. & Richter, C. Autofluorescence of living cells. *Journal of microscopy* **191**, 1-7 (1998).
